# Emissions intensity and carbon stocks of a tropical Ultisol after amendment with Tithonia green manure, urea and biochar

**DOI:** 10.1016/j.fcr.2017.05.013

**Published:** 2017-08-01

**Authors:** Bernard Fungo, Johannes Lehmann, Karsten Kalbitz, Moses Tenywa, Margaret Thionģo, Henry Neufeldt

**Affiliations:** a National Agricultural Research Organization (NARO), P.O. Box 1752, Kampala, Uganda; b Institute for Biodiversity and Ecosystem Dynamics (IBED), Faculty of Science, University of Amsterdam, Science Park 904, 1098 XH Amsterdam, The Netherlands; c CGIAR Research Program on Climate Change, Agriculture and Food Security (CCAFS), World Agroforestry Center (ICRAF), P.O. Box 30667, UN, Avenue-Gigiri, Nairobi, Kenya; d Soil and Crop Sciences, Cornell University, Bradfield Hall, Ithaca, NY 14853, USA; e Soil Resources and Land Use, Institute of Soil Science and Site Ecology, Dresden University of Technology, Pienner Strasse 19, 01737 Tharandt, Germany; f Collage of Agricultural and Environmental Sciences, Makerere University, P.O. Box 7062, Kampala, Uganda

**Keywords:** Biochar, Greenhouse gasses, Emission Intensity, Carbon cycling, Ultisol

## Abstract

Biochar has been shown to reduce soil emissions of CO_2_, CH_4_ and N_2_O in short-term incubation and greenhouse experiments. Such controlled experiments failed to represent variable field conditions, and rarely included crop growth feedback. The objective of this study was to assess the effect of biochar, in comparison to green manure and mineral nitrogen, on greenhouse gas Emissions Intensity (EI = emissions in CO_2_ equivalents per ton of grain yield) in a low-fertility tropical Ultisol. Using a field trial in western Kenya, biochar (0 and 2.5 t ha^−1^; made from Eucalyptus wood) was integrated with urea (0 and 120 kg N ha^−1^) and green manure (*Tithonia diversifolia*; 0, 2.5 and 5 t ha^−1^) in a factorial design for four consecutive seasons from October 2012 to August 2014. Compared to the control, biochar increased soil CO_2_ emissions (9–33%), reduced soil CH_4_ uptake (7–59%) and reduced soil N_2_O emissions (1–42%) in each season, with no seasonal differences. N_2_O emissions increased following amendment with *T. diversifolia* (6%) and urea (13%) compared to the control. Generally, N_2_O emissions decreased where only biochar was applied. The greatest decrease in N_2_O (42%) occurred where all three amendments were applied compared to when they were added separately. EI in response to any of the amendments was lower than the control, ranging from 9 to 65% (33.0 ± 3.2 = mean ± SE). The amendments increased SOC stocks by 0.1–1.2 t ha^−1^ year^−1^ (mean ± SE of 0.8 ± 0.09 t ha^−1^ year^−1^). The results suggest decreased net EI with biochar in low fertility soils mainly through greater net primary productivity (89% of the decrease).

## Highlights

• Biochar increased CO_2_ emissions, reduced CH_4_ soil uptake, and reduced N_2_O emissions.
• N_2_O decreased the most (42%) where all the three amendments were present.
• Decreased in EI with biochar in low fertility soils is mainly through greater net primary productivity.
• Biochar alone decreased SOC but increase it when applied together with urea and Tithonia.

## 1 Introduction

The search for climate-smart agricultural production technologies is directing research to identify innovations that address multiple benefits such as crop productivity, carbon sequestration and mitigation of soil-atmosphere greenhouse gas (GHG) emissions. Addition of biochar (pyrogenic organic matter) to agricultural soils as a management strategy has reportedly increased crop yields in several studies but has shown variable effects on GHG fluxes (Knoblauch et al., 2011, Cayuela et al., 2014). Biochar may affect fluxes of GHGs such as CO_2_, CH_4_ and N_2_O by a variety of mechanisms, including: (i) the turnover rate of soil organic matter (SOM), which in turn determines the availability of C and N, the precursors for GHG production or consumption, (ii) soil physical properties (e.g. gas diffusivity, aggregation, water retention) (Quin et al., 2014); (iii) soil chemical properties (e.g. pH, Eh, availability of organic and mineral N and dissolved organic C, organo- mineral interactions); and (iv) soil biological properties (e.g. microbial community structure, microbial biomass and activity, macro-fauna activity, N cycling enzymes) (Van Zwieten et al., 2010).

Biochar may also change the effects of adding easily mineralizable organic matter as well as mineral N fertilizers on GHG emissions from soil. Additions of legume materials as a fertilizer provide both N and C that typically lead to greater GHG emissions from soils (Gentile et al., 2009). Similarly, the amount of fertilizer N additions is considered proportional to the N_2_O emissions (Manzoni and Porporato, 2009, Mori and Hojito, 2011). It is not clear, if simultaneous addition of biochar with either fertilizer N or legume mulch or a combination if the two may result in GHG emission reductions. Uncertainty also exists whether any emission reductions would persist over several cropping seasons.

It is not clear what role possible feedback through enhanced crop growth plays to the GHG budget. Greater crop growth and presumably greater C return to soil have been found where the pH is increased by biochar to neutral values (Jeffery et al., 2015) and this feedback would therefore be expected to be greatest in acid tropical soils. Whereas Spokas (2013) suggested that biochar has mainly shorter-term GHG mitigation effect (few days to several weeks) after application, Lentz et al. (2014) indicated that the effects may be long-lived. As such, questions remain concerning the long-term implications in cropping periods particularly for field-based biochar studies. Zimmerman et al. (2011) observed that native SOM mineralization was higher during the early incubation stage (first 90 days) and low during the later incubation stage (250–500 days). Maestrini et al. (2014) also reported pyrogenic OM (PyOM) to have promoted native OM mineralization during the first 18 days and inhibited it afterward (up to 150 days).

The objectives of this study were to determine the effect of biochar on (i) GHG fluxes (CO_2_, CH_4_ and N_2_O), (ii) Emissions Intensity (EI; the net CO_2_-equivalent for CH_4_ and N_2_O per ton of grain yield), and (iii) changes in soil organic carbon (SOC) and ecosystem carbon balance of a low-fertility tropical agricultural soil when integrated with organic and mineral N inputs. The overall hypothesis is that biochar is responsible for controlling the release of labile N and C from high N mineral and organic amendments and an accompanying reduction in CO_2_, CH_4_ and N_2_O emissions. Specifically, we hypothesize that compared to un-amended soil, biochar (i) reduces availability of N from both organic and mineral sources such as *T. diversifolia* and urea to thereby reduce N_2_O emission resulting from interactions of N_2_O with biochar; (ii) increases availability of easily mineralizable C from both soil and organic amendment to reduce the CH_4_ soil sink; (iii) affects emissions of CH_4_ and N_2_O early on, but not in later seasons as active surfaces get saturated with time; and (iv) increases plant growth as a result of biochar additions that are more important than changes in other soil processes affecting GHG emissions.

## 2 Materials and methods

### 2.1 Study site

The field experiment was established in September 2012 at Kapsengere on the southern Nandi hills in western Kenya (00′ 09′ 34″N and 34′ 57′ 37″E). The site receives ∼2000 mm mean annual rainfall in a bimodal distribution, with two growing seasons per year, March–July and September–January. The mean annual temperature is 26 °C. The soils are classified as Typic Kandiudults (USDA, 1999) developed on biotite-gneiss parent material. The experimental field was divided into three blocks. Soil properties before the experiment were determined by taking two samples from each block (six composite samples in total). The composite sample was obtained by mixing soil taken at four random locations. These were assumed to adequately represent the entire field where the experiment was established. The soil samples were analyzed using methods described in Fungo et al. (2014); in addition, particle size distribution was determined by the hydrometer method (Soil Texture Unit 1067; LaMotte Co., Chestertown, MD, USA) (soil properties in Table 1). The natural vegetation is composed of tropical rainforest of the Guineo-Congolian type. The experiment was conducted for four consecutive maize growing seasons: September–December 2012; March–August 2013; September–December 2013; March–August 2014. The seasons are henceforth referred to as Short Rains 2012 (SR2012); Long Rains 2013 (LR2013); Short Rains 2013 (SR2013) and Long Rains 2014 (LR2014), respectively.

**Table 1:** Physical-chemical properties of the soil (0–0.2 m) and the amendments used in the field trial in western Kenya (n = 6 replicates for soil; triplicate measurements for amendments; means with standard errors in brackets).

Property	Biochar		Soil		Green manure (T. diversifolia)		
					Property		
pH	6.3	(0.1)	6.0	(0.1)	N (mg kg^−1^)	21.5	(0.5)
C (g kg^−1^)	868	(11)	23.3	(0.1)	P (mg kg^−1^)	2.3	(0.1)
N (g kg^−1^)	27.0	(0.9)	21.0	(0.9)	K (mg kg^−1^)	43.2	(1.2)
P (mg kg^−1^)	135	(3.7)	9.30	(0.2)	Ca (mg kg^−1^)	13.6	(0.2)
K (mg kg^−1^)	1490	(14)	223	(10)	Na (mg kg^−1^)	72.7	(0.9)
Ca (mg kg^−1^)	1920	(17)	1950	(10)	Fe (mg kg^−1^)	951	(10)
Na (mg kg^−1^)	180	(7.3)	15.9	(0.6)	Zn (mg kg^−1^)	89.7	(1.6)
Mg (mg kg^−1^)	150	(4.5)	312	(9.4)	Mg (mg kg^−1^)	2.6	(0.0)
Al (mg kg^−1^)	559	(9.8)	939	(16)	S (mg kg^−1^)	2.5	(0.0)
S (mg kg^−1^)	36.5	(1.4)	14.0	(0.8)	Mn (mg kg^−1^)	264	(5)
Fe (mg kg^−1^)	164	(5.7)	67.2	(1.6)	Cu (mg kg^−1^)	11.0	(0.2)
Zn (mg kg^−1^)	108	(2.4)	13.5	(0.4)	B (mg kg^−1^)	53.2	(1.6)
Mn (mg kg^−1^)	188	(4.9)	782	(14)	Mo (mg kg^−1^)	1.3	(0.0)
Cu (mg kg^−1^)	0.77	(0.1)	1.97	(0.1)			
B (mg kg^−1^)	1.07	(0.0)	0.33	(0.0)			
C.E.C (meq 100 g^−1^)	18.2	(0.6)	16.2	(0.5)			
EC (S mm^−1^)	196	(6.5)	88.0	(1.2)			
Silt (%)	nd		17.5	(0.3)			
Sand (%)	nd		10.7	(0.4)			
Clay (%)	nd		71.6	(2.0)			

nd = not determined.

### 2.2 Biochar and green manure

Biochar was produced from eucalyptus wood by chopping and grinding to pass through a 2-mm sieve. The ground material was pyrolyzed to a maximum temperature of 550 °C using a thermostat-regulated kiln with continuous agitation to provide homogeneous charring conditions and retained at this temperature for one hour before cooling to room temperature. Green manure from *T. diversifolia* was prepared by cutting leaves from the field and chopping them into 50-mm lengths, air-drying and grinding to pass through a 2-mm sieve before field application. The physical and chemical characteristics of the soil were analyzed following the same procedures as in Fungo et al. (2014) and are presented in Table 1.

### 2.3 Experimental design

The experiment was laid out in a randomized complete block design with three replicates. Treatments included the following: two levels of biochar (0 and 2.5 t ha^−1^); three levels of *T. diversifolia* green manure (0, 2.5 and 5 t ha^−1^); and two levels of urea application (0 and 120 kg N ha^−1^) in a full factorial design (Table 2). Treatments were indicative of the range of conventional management practices of many small-holder farmers in integrated soil fertility management systems. Each treatment was established in 2 × 2-m plot separated by a one meter distance within and between rows.

**Table 2:** Experimental treatments for determining the effect of biochar, T. diversifolia green manure and urea on fluxes of CO_2_, CH_4_ and N_2_O in a maize field in western Kenya. Biochar was applied only once at the start of the experiment while urea and tithonia were applied every season for four consecutive seasons.

Treatment	Biochar		*T. diversifolia*		Mineral N (Urea)	
	Rate (t ha^−1^)	*Code*	*Rate* (t ha^−1^)	Code	Rate (kg N ha^−1^)	Code
1 (B_0_T_0_U_0_)(Control)	0	B0	0.0	T0	0	U0
2 (B_0_T_2.5_U_0_)	0	B0	2.5	T2.5	0	U0
3 (B_0_T_5_U_0_)	0	B0	5.0	T5	0	U0
4 (B_0_T_0_U_120_)	0	B0	0.0	T0	120	U120
5 (B_0_T_2.5_U_120_)	0	B0	2.5	T2.5	120	U120
6 (B_0_T_5_U_120_)	0	B0	5.0	T5	120	U120
7 (B_2.5_T_0_U_0_)	2.5	B2.5	0.0	T0	0	U0
8 (B_2.5_T_2.5_U_0_)	2.5	B2.5	2.5	T2.5	0	U0
9 (B_2.5_T_5_U_0_)	2.5	B2.5	5.0	T5	0	U0
10 (B_2.5_T_0_U_120_)	2.5	B2.5	0.0	T0	120	U120
11 (B_2.5_T_2.5_U_120_)	2.5	B2.5	2.5	T2.5	120	U120
12 (B_2.5_T_5_U_120_)	2.5	B2.5	5.0	T5	120	U120

### 2.4 Management of experiment

Precipitation and air temperature were monitored throughout the experiment with the help of a weather station on site. Application of biochar was done only once at the start of the first season in October 2012. The same amounts of green manure (2.5 or 5.0 t ha^−1^), were applied to each plot once at the start of each season (four applications in total). Mineral N (Urea; 261 kg ha^−1^) was applied in two splits at a total of 120 kg N ha^−1^ per season; 40% at planting and 60% at 30 days-after planting. Due to the inherently low fertility of the soil, 30 kg ha^−1^ of P as Triple Super Phosphate (TSP) (55 kg P_2_O_5_ ha^−1^) and 30 kg ha^−1^ of K as Muriate of Potash (MoP) (45 kg K_2_O ha^−1^) were applied to each plot at the start of each season. The amendments were applied by broadcasting on the soil surface by hand and immediately incorporating into the 0.1 m top soil. In plots where the combinations were applied, biochar was applied first, followed by Tithonia and then urea. Two seeds of a maize cultivar HB 513 were planted at the start of every season at a spacing of 0.25 m within and 0.5 m between rows (40 plants per plot). Weeding was done at 30 and 50 days after planting using a hand hoe. Thinning was done during the first weeding to retain one plant per planting hole.

### 2.5 Gas measurements

Measurements of CO_2_, CH_4_ and N_2_O fluxes were conducted using a static closed chamber method (Neftel et al., 2006; Morris et al., 2013). The chamber consisted of a PVC tube (diameter = 0.3 m; height = 0.15 m) transversely divided into two parts to make a base (0.05 m) and a cover (0.1 m). The base was driven into the soil to reach ∼0.02 m below the soil surface. To ensure air-tight conditions, a rubber ring was placed between the base and the cover. A photo-acoustic infrared field gas monitor (INNOVA 1402, Lumasense Technologies A/S, Ballerup, Denmark) was used to determine the gas concentrations. The accuracy and precision of standard gas concentration measurements with Photo Acoustic Spectroscopy (PAS) and Gas Chromatography (GC) have been shown to be comparable (Jung et al., 2012, Zhao et al., 2012, Iqbal et al., 2013). The gas monitor was connected to the chamber by two 0.7 m-long teflon tubes as gas inlet and outlet. Inside the cuvette, air humidity and temperature were monitored by a digital thermo-hygrometer (PCE–313 A, Paper-Consult Engineering Group, Meschede, Germany) attached to the cover from the outside while only the sensor reached inside the chamber through a rubber screw connector. Two chambers, marked “1” and “2”, were set up in each plot. All chambers marked “1” in all the treatments were sampled on day 1 and those marked “2” were sampled the following day. The values for chamber 1 and 2 for each plot were then averaged. For each gas sampling event INNOVA recorded four measurements at 2-min intervals after closing the chamber. Flux measurements were conducted weekly except during dry periods where bi-monthly measurements were taken. Fluxes are generally constant during dry, low moisture soil conditions. A total of 53 data points were obtained and used in the analysis.

### 2.6 Harvesting and yield determination

Above ground biomass was considered as the sum of stover, cobs and grain. Corn ears from each plot were removed from their shucks 120 days after planting, bagged, and dried for six days in a shed. Stover in each plot was cut ∼0.02 m above the soil surface, weighed and dried in a shed for six days. All above ground residues from the plot were never returned to the plot in order to determine how much of the SOC could be credited to above ground vs. below ground inputs. After drying, ears were mechanically shelled, and cob and kernel biomass was determined for each plot. Moisture content in the samples was determined by taking biomass of five fresh randomly selected plants to the oven for 48 h at 60 °C. Soil samples were taken from a 0–0.2 m depth at the beginning and end of the experimental period. For the soil samples, total organic carbon (TOC) was determined by the dry combustion method (Elementar Vario EL, Hanau, Germany) and assumed that TOC = SOC since these acid soils have negligible amounts of carbonates. The available phosphate content was determined using the Lancaster method (RDA, 1988). Exchangeable cations were estimated using Inductively Coupled Plasma Spectroscopy (OPTIMA 4300DV, Perkin Elmer, USA) after extraction with 1N ammonium acetate (pH 7.0) using a soil:water ratio of 1:20 w/v.

### 2.7 Data analysis

#### 2.7.1 Data management

Cumulative gas fluxes were obtained by calculating the area under the flux-time curve and summing the results while assuming linear changes in measurements between time intervals. Global Warming Potential (GWP, the sum of cumulative gas emissions of CH_4_ and N_2_O, multiplied by the radiative forcing factor of each gas 25 and 298, respectively, for a time horizon of 100 years) (USEPA, 2007) calculated using the following equation:

GWP = 25 × (E-CH_4_) + 298 × (E-N_2_O)

Where, GWP is the emission in CO_2_-equivalents per hectare, E-CH_4_ and E-N_2_O are the emissions of CH_4_ and N_2_O per hectare during a given year, respectively. Emissions Intensity (EI) was then obtained by dividing the cumulative CO_2_-equivalent of the gas fluxes by the cumulative grain yield of each treatment over the experimental period.

The cumulative emission for each treatment was derived using a linear trapezoidal rule with sampling dates as the time intervals. For seasonal comparisons, the cumulative flux was restricted to the 120 days between planting and harvesting. Changes in SOC stock were calculated as the difference between values at the beginning and end of the experiment, after subtracting the C addition from biochar and *T. diversifolia*.

#### 2.7.2 Statistical analysis

Differences in the net EI for each treatment were calculated as the difference between the treatment value and that of the control. Data for CO_2_ was normally distributed and did not require transformation but CH_4_ and N_2_O data were natural log-transformed before ANOVA. Treatment effects and their interaction were examined with repeated-measures ANOVA using comparisons with seasons. A fixed-effect model in Stata 12 (StataCorp LP, 4905 Lakeway Drive, College Station, Texas 77845 USA) was used with a nested design with biochar and Tithonia within urea. Post hoc separation of means was done using Least Significant Difference (LSD) at a 5% level of significance using the Stata 12 statistical software.

## 3 Results

### 3.1 Dynamics of GHG fluxes

#### 3.1.1 Daily dynamics

**Fig. 1:**
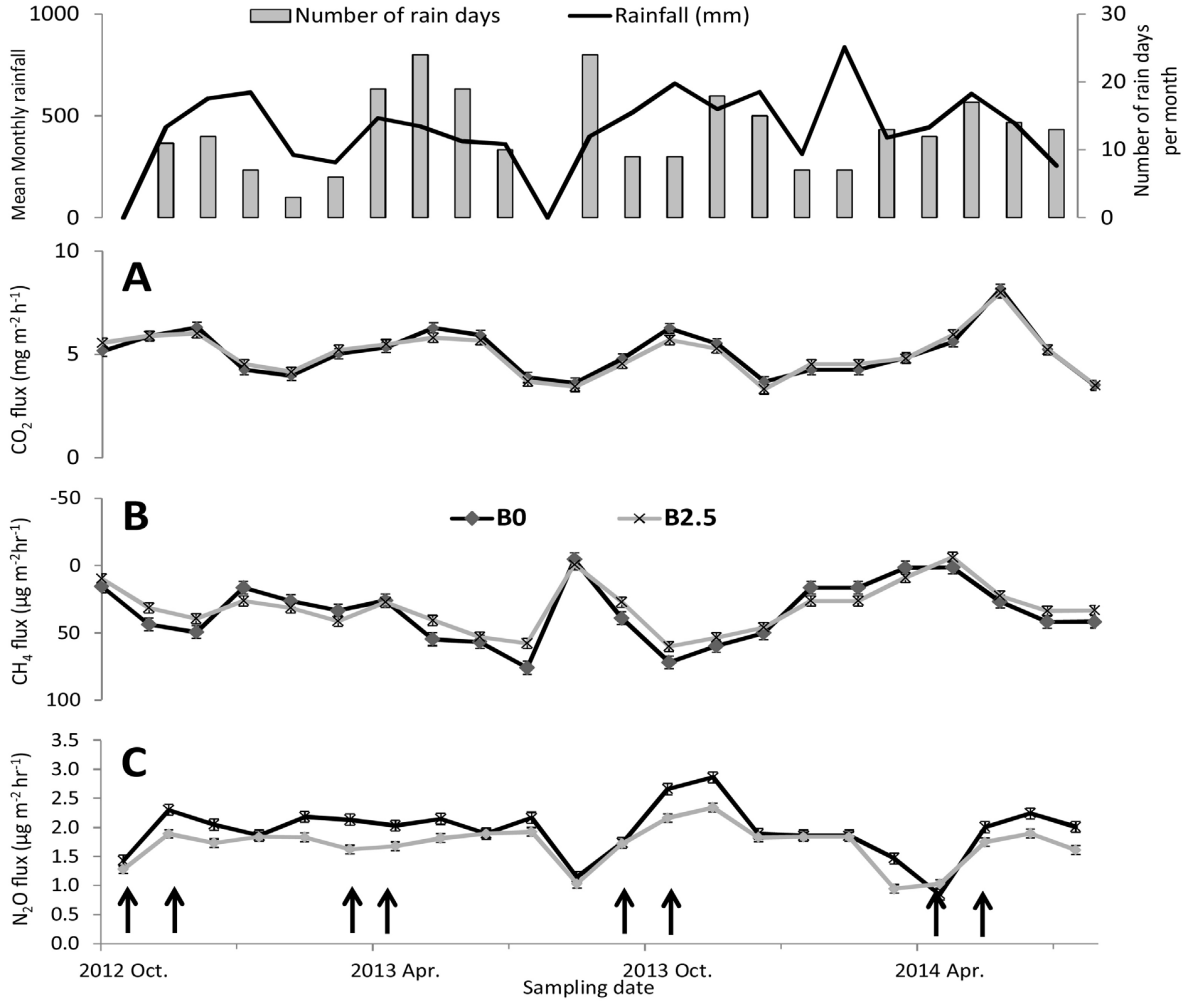
Weather patterns (top graph), CO_2_ (A), CH_4_ (B) and N_2_O (C) fluxes during four seasons of growing maize in western Kenya after amendment with mineral fertilizer (urea, 120 kg N ha^−1^) and green manure (T. diversifolia, 2.5 and 5.0 t ha^−1^) and biochar (0 or 2.5 t ha^−1^). Error bars are standard errors. Dates on the x-axis indicate the planting time. n = 3. Arrows at the x-axis show the dates when urea was applied.

The mean daily fluxes of CO_2_, CH_4_ and N_2_O were 5.4 mg m^−2^ h^−1^, 39 μg m^−2^ h^−1^and 1.96 μg m^−2^ h^−1^, respectively (Fig. 1). Emissions closely followed weather patterns with higher CO_2_ and N_2_O emissions as well as lower CH_4_ uptake during the wet seasons. During the dry seasons, fluxes of CH_4_ and N_2_O were generally below average. Variability in daily fluxes, as expressed by the coefficient of variation (CV), was generally higher for CH_4_ (60%) compared to CO_2_ and N_2_O (21%). Biochar effects were not observable for daily CO_2_ measurements, but its effect was observed for CH_4_ and N_2_O, where it generally reduced the CH_4_ sink capacity of the soil and reduced N_2_O emission.

#### 3.1.2 Seasonal dynamics

**Fig. 2:**
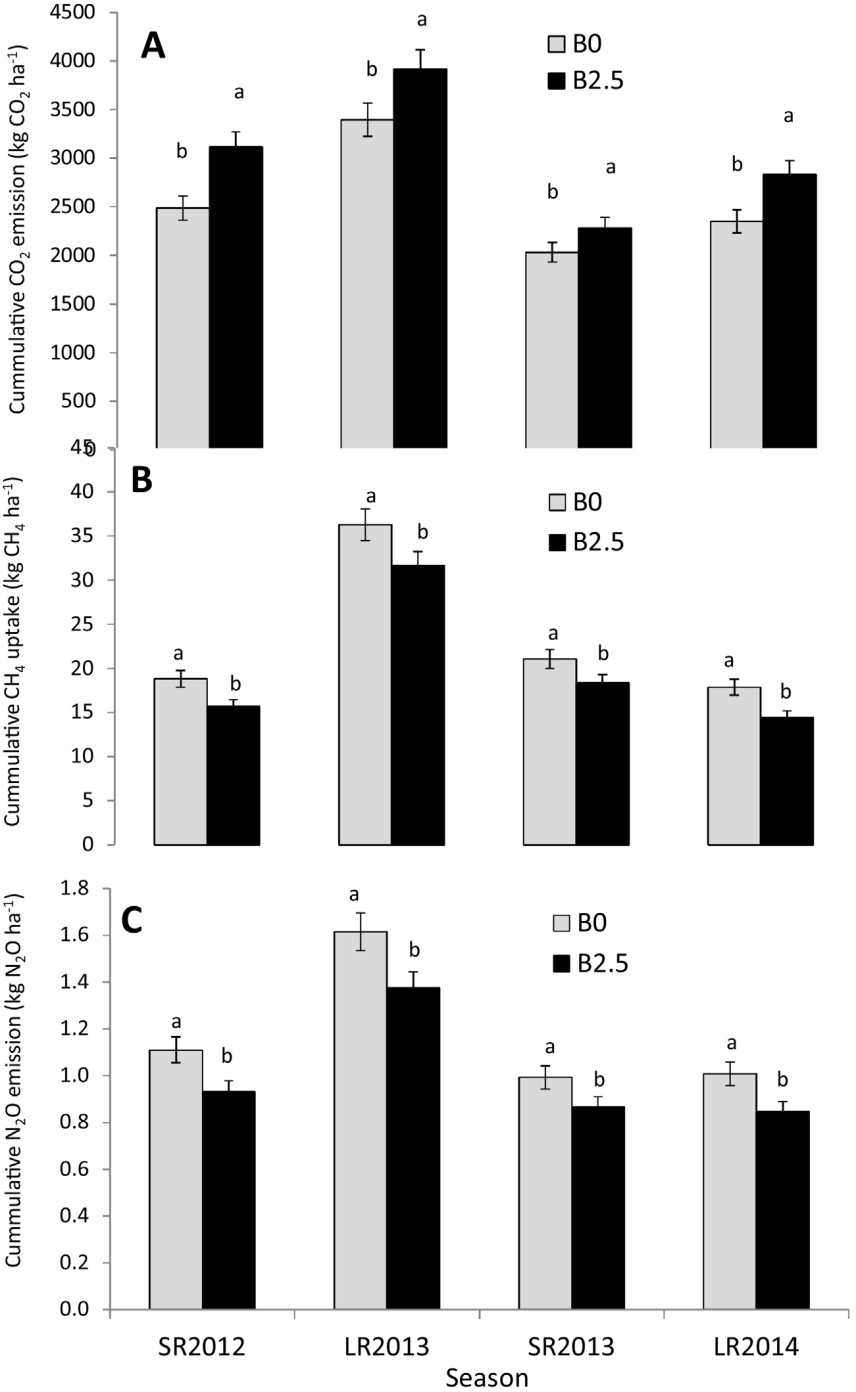
Seasonal cumulative CO_2_, (A) emission, CH_4_ uptake (B), and N_2_O emission (C) fluxes during four-season maize trial in western Kenya after amendment with mineral fertilizer (urea) and green manure (T. diversifolia) and biochar. Within each cluster, bars with different letters are significantly different. Error bars are standard errors, n = 3.

Seasonal cumulative CO_2_ emission increased by 17% (Fig. 2A) throughout the four seasons. CH_4_ uptake was reduced by 17% (Fig. 2B) in biochar-amended compared to control plots, and this reduction was observed in all the four seasons. The decrease in CH_4_ uptake due to biochar was maintained in all the seasons, but there was no significant difference in CH_4_ uptake among the seasons. Among all the seasons, LR2013 experienced the highest cumulative uptake of CH_4_. Consistent 15% reduction of N_2_O emissions were observed due to biochar additions, irrespective of season (Fig. 2C). Similar to CH_4_, LR2013 experienced the highest cumulative emission of N_2_O. Emission of N_2_O in this season was higher (P = 0.03) than those of all the other seasons likely because the rains were highest in this season.

#### 3.1.3 Annual GHG emissions

All the three amendments, except urea (which had no effect on CH_4_), affected each of the GHGs (Table 3). Annual flux of CO_2_ was increased by all amendments (Table 3).

**Table 3:** Three-way ANOVA for the effects of biochar, T. diversifolia green manure and urea on fluxes of CO_2_, CH_4_ N_2_O, GWP, maize grain yield and EI in maize field in western Kenya. P-values in bold show means that were significantly different p = 0.05, n = 3.

Source of variation	CO_2_		CH_4_		N_2_O		GWP		Grain Yield		EI	
	F	P	F	P	F	P	F	P	F	P	F	P
Biochar	6.11	**0.021**	6.29	**0.026**	5.65	**0.011**	12.5	**0.007**	10.5	**0.031**	10.6	**0.010**
*T. diversifolia*	12.25	**0.000**	3.46	**0.047**	5.02	**0.015**	10.4	**0.025**	1.2	0.96	12.0	**0.017**
Urea	8.62	**0.007**	3.01	0.095	8.43	**0.006**	8.0	**0.045**	11.4	**0.034**	18.0	**0.000**
Biochar *x T. diversifolia*	1.27	0.271	1.13	0.299	0.88	0.358	16.7	**0.000**	10.8	**0.031**	9.3	**0.021**
Biochar *x* Urea	1.98	0.160	1.13	0.339	4.96	**0.037**	11.2	**0.016**	10.1	**0.038**	15.2	**0.000**
*T. diversifolia x* Urea	1.67	0.208	1.04	0.370	1.27	0.098	0.0	0.095	0.0	**0.095**	11.2	**0.018**
Biochar *x T. diversifolia x* Urea	1.36	0.276	1.61	0.220	1.17	0.325	10.8	0.031	4.9	**0.042**	4.3	**0.047**

Bold numbers show significant effect at 95% level of confidence.

The increase in CO_2_ emissions ranged from 6 to 33%. The highest increase was observed where both *T. diversifolia* and urea were present (P = 0.02). On average, amendments of biochar, tithonia or urea increased CO_2_ emissions by 10% (P = 0.034). No interaction between either biochar and *T. diversifolia* or biochar and urea was observed (P > 0.05). Also, no effect of increasing *T. diversifolia* from 2.5 to 5.0 t ha^−1^ in terms of CO_2_ emissions was observed.

Both biochar and *T. diversifolia* reduced the soil uptake of CH_4_, while urea had no effect. Reduction in soil CH_4_ uptake ranged from 7 to 59% (Table 4). There was no interaction between amendments, and the level of *T. diversifolia* additions on CH_4_ uptake between the 2 and 4 t ha^−1^ application rates. The largest reduction in CH_4_ uptake was observed when biochar, tithonia and urea were added together at the highest quantities. CH_4_ uptake decreased progressively as more amendments were added.

**Table 4:** Annual grain yield (two seasons), CH_4_ and N_2_O emissions, GWP (calculated only from CH_4_ and N_2_O) and EI of maize production over four consecutive seasons under biochar, *T. diversifolia* and urea amendment. Means (±S.E.) in the same column followed by different letters are significantly different at p < 0.05, n = 3.

Treatment	CO_2_ (t ha^−1^ yr^−1^)		CH_4_ uptake (t ha^−1^ yr^−1^)		N_2_O emission (t ha^−1^ yr^−1^)		GWP (t CO_2_-eq ha^−1^ yr^−1^)		Maize Grain yield (t ha^−1^ yr^−1^)		EI (t CO_2_-eq t^−1^ grain yr^−1^)	
1 (B_0_T_0_U_0_)	3.7	(±0.6)d	0.061	(±0.017)a	0.003	(±0.009)a	0.67	(±0.5)a	14.8	(±1.0)f	0.05	(±0.01)a
2 (B_0_T_2.5_U_0_)	4.9	(±0.1)a	0.057	(±0.023)b	0.003	(±0.002)a	0.57	(±0.34)b	15.1	(±0.6)f	0.04	(±0.03)b
3 (B_0_T_5_U_0_)	4.8	(±0.3)a	0.058	(±0.006)b	0.003	(±0.006)a	0.59	(±0.18)b	14.9	(±0.1)f	0.04	(±0.02)bc
4 (B_0_T_0_U_120_)	4.1	(±0.6)ca	0.057	(±0.007)b	0.003	(±0.004)a	0.60	(±0.11)b	15.9	(±0.8)e	0.04	(±0.02)c
5 (B_0_T_2.5_U_120_)	5.0	(±0.1)a	0.052	(±0.009)bc	0.003	(±0.007)a	0.47	(±0.04)c	18.4	(±1.4)c	0.03	(±0.03)c
6 (B_0_T_5_U_120_)	4.9	(±0.1)c	0.053	(±0.010)bc	0.002	(±0.009)b	0.59	(±0.22)b	19.1	(±0.1)b	0.03	(±0.05)d
7 (B_2.5_T_0_U_0_)	4.0	(±0.6)b	0.052	(±0.018)bc	0.002	(±0.005)b	0.59	(±0.32)b	12.8	(±1.8)g	0.05	(±0.01)a
8 (B_2.5_T_2.5_U_0_)	4.4	(±0.3)a	0.050	(±0.006)c	0.003	(±0.005)a	0.42	(±0.06)d	12.6	(±1.8)g	0.03	(±0.06)d
9 (B_2.5_T_5_U_0_)	5.0	(±0.1)b	0.046	(±0.029)d	0.002	(±0.001)b	0.47	(±0.18)d	17.4	(±1.6)d	0.03	(±0.01)e
10 (B_2.5_T_0_U_120_)	4.6	(±0.6)b	0.047	(±0.014)d	0.002	(±0.007)b	0.44	(±0.36)cd	19.8	(±1.0)a	0.02	(±0.03)f
11 (B_2.5_T_2.5_U_120_)	4.6	(±0.9)b	0.055	(±0.025)b	0.003	(±0.001)ab	0.58	(±0.34)b	17.0	(±3.2)d	0.03	(±0.01)cd
12 (B_2.5_T_5_U_120_)	4.4	(±0.1)b	0.034	(±0.020)e	0.002	(±0.007)c	0.26	(±0.31)e	16.9	(±0.8)d	0.02	(±0.03)g

Cumulative N_2_O emissions progressively decreased by up to 42% (mean ± SE of 19 ± 4.1%) where biochar was amended but did not change where *T. diversifolia* or urea were added on their own compared to an un-amended control (Table 4). Biochar-induced decreases in N_2_O emission were greatest (42%) where *T. diversifolia* and urea were both present compared to when they were separately applied. N_2_O emissions with biochar + urea additions were significantly lower than those with urea alone (Table 4). The interaction between biochar and *T. diversifolia* was significant only at the higher level of *T. diversifolia.*

### 3.2 Grain yield

Maize grain yields ranged from 12.6 to 19.8 t ha^−1^ yr^−1^ (mean = 16.4 t ha^−1^ yr^−1^), corresponding to a decrease of 15% and increase of 34%, respectively, compared to the control. Decreases in maize grain yield were observed where biochar was applied either alone or in combination with low amounts of *T. diversifolia* (B_2.5_T_0_U_0_, −15%, and B_2.5_T_2.5_U_0_, −14%, respectively). High amounts of *T. diversifolia* (T_5_) significantly increased yields by 9%. The greatest increase in maize grain yield was observed where biochar was combined with urea (B_2.5_T_0_U_120_, 34%). This was followed by yields where high amounts of *T. diversifolia* (5 t ha^−1^) were jointly applied with urea (treatment B_0_T_5_U_120_ at 29%) (Table 4). Grain yield of the combination of the three amendments was higher than either biochar or *T. diversifolia* applied alone (Table 4). Grain yield comprised of 35% (SD = 0.03) of total above ground biomass and this proportion was comparable for all treatments.

### 3.3 Global worming potential (GWP)

Biochar when applied alone caused a significantly lower GWP (8% or 6.3 ± 0.01 t CO_2_-eq ha^−1^ yr^−1^) compared to the control (Table 4). GWP caused by additions of *T. diversifolia* and urea compared to no additions (control) significantly increased by 32% and 12% corresponding to an increase by 7.7 ± 0.8 and 6.5 ± 0.6 t CO_2_-e ha^−1^ yr^−1^, respectively. *T. diversifolia* or urea additions resulted in significantly higher yield compared to the control, but were not significantly different from each other. Grain yield was significantly higher with the sole biochar addition. The interactive effect between biochar and urea increased yield significantly but no difference in yield was observed where tithonia and urea were applied together or where biochar was applied with tithonia. In a combined application of biochar, urea and *T. diversifolia*, doubling the amounts of *T. diversifolia* halved the GWP. The difference between GWP as a result of biochar + *T. diversifolia* and biochar + urea additions was significantly lower than the difference in the respective sole applications of *T. diversifolia* versus urea.

### 3.4 Emissions intensity (EI)

Biochar and tithonia applied alone caused significantly higher EI compared to the control, but EI as a result of urea additions was not significantly different from that of the control. There was no interactive effect between tithonia and urea application with respect to EI. Except for the interaction of 2.5 t biochar ha^−1^ and urea (B_2.5_T_0_U_120_ in Table 4), all the other treatments showed significantly higher EI than the control (*p* *=* *0.035*), with increases ranging from 8 to 39%. Apart from the control, the highest EI corresponded with additions of only *T. diversifolia* green manure (B_0_T_2.5_U_0_ and B_0_T_5_U_0_) or urea additions (B_0_T_0_U_120_). The lowest EI was observed where biochar and urea were added without or with high *T. diversifolia*. As *T. diversifolia* green manure additions increased, the EI correspondingly decreased but only in the presence of biochar. The decrease in EI with greater additions of *T. diversifolia* corresponded to increases in CO_2_ emission by 14% and decreases in N_2_O emission by 16% (Table 4).

### 3.5 Changes in SOC stocks

**Fig. 3:**
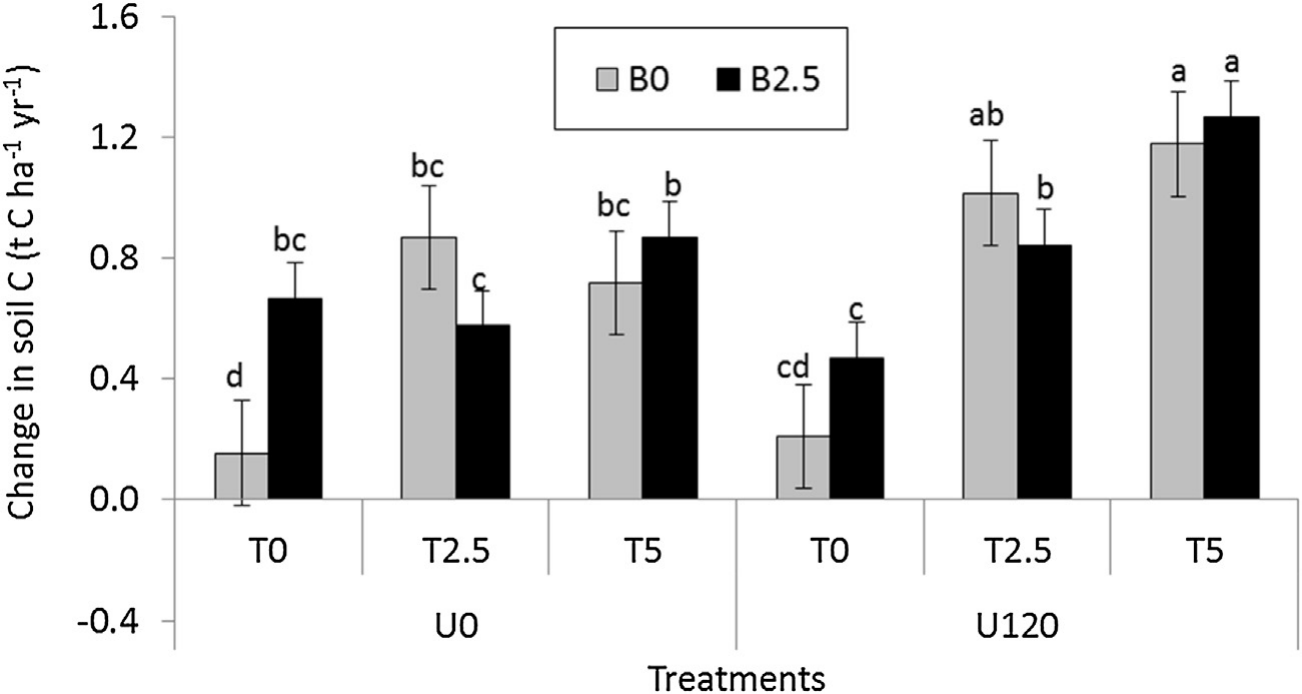
Annual changes in soil organic C stock (top 0.15 m) under biochar (B in t ha^−1^), *T. diversifolia* (T in t ha^−1^) and urea (U in kg N ha^−1^) amendments compared to the control. Means (±S.E.) followed by different letters are significantly different at p < 0.05, n = 3.

Amendments increased SOC stocks in the range of 0.7–7.1%, corresponding to 0.1–1.2 t C ha^−1^ year^−1^ (mean ± SE of 0.8 ± 0.09 t C ha^−1^ year^−1^). Except for the control (where no amendment was applied), which showed no significant difference in SOC over time, all the other treatments showed gains in SOC (Fig. 3). The smallest increment (0.2 ± 0.01 t C ha^−1^ yr^−1^) was observed without any additions while the highest increase (1.2 ± 0.04 t C ha^−1^ yr^−1^) was recorded where urea was applied with *T. diversifolia* irrespective of biochar additions. The increase in SOC stocks due to biochar or *T. diversifolia* additions was comparable (0.7 ± 0.02 t C ha^−1^ yr^−1^, or 2.8%) to the control. Soil OC stocks in response to additions of 2.5 t ha^−1^ of tithonia increased by 27% compared to the control, and was comparable to additions of 5 t ha^−1^ of tithonia. Additions of 2.5 t ha^−1^ of tithonia together with urea increased SOC by 39% compared to the control.

## 4 Discussion

### 4.1 Dynamics of GHGs

Stimulation of CO_2_ emissions following biochar addition observed in this study (Figs. 1A and 2A) concurs with what has been observed in previous studies and has been attributed to the supply of easily mineralizable C and improvement in soil physical properties for microbial activity (Lehmann et al., 2011; Sagrilo et al., 2014) and increased root respiration (Major et al., 2010). However, reduction in CO_2_ emission after biochar addition has also been reported in incubations without plants, and is usually associated with limited N supply due to N immobilization by amended biochar (Laird et al., 2010, Wang et al., 2015). In our case, the amount of biochar was relatively low (0.2% compared to the average 2% w/w in most studies) and could not immobilize significant amounts of soil N while crop growth significantly increased, hence the consistent increase in CO_2_ (that included root respiration) with all biochar plots. The lack of an interactive effect between biochar and tithonia as well as urea and tithonia with respect to CO_2_ emission (Table 3) was possibly due to the fact that sufficient C was supplied by biochar or *T. diversifolia* making further inputs temporarily unnecessary for microbial use. Contrary to our findings, Rogovska et al. (2011) found reduced CO_2_ emission after 500 days of incubation for a green manure-biochar mixture. The authors attributed this observation to the fact that biochar stabilized green manure C by influencing biochemical recalcitrance or physical protection of green manure C (Krull et al., 2003), or that green manure additions reduced the ability of biochar to enhance mineralization of soil organic matter. Our results did not show interactions between biochar and green manure possibly because the mixing of the two amendments was not as uniform as it was in the incubation of Rogovska et al. (2011). Whereas biochar largely remains unchanged in space, tithonia and urea may, after incorporation into the soil, leach to lower soil layers. The subsequent reactions of these amendments may thus remain independent. Some studies on placement of amendments may elucidate this notion. It is important to note that CO_2_ can be produced by both enhance mineralization of soil organic matter or biochar itself (Wardle et al., 2008). Our study measured only soil-atmosphere fluxes of CO_2_, and could not attribute the increase to a particular CO_2_ production process. Nonetheless, the fact that there was an increase in SOC with biochar-tithonia additions (Fig. 3) suggest an overall net positive balance towards soil C sequestration. Future studies should estimate the net effect of biochar application on soil respiration and biochar mineralization. Life cycle assessment to determine C sequestration including CO_2_ emitted during biochar production will also be relevant.

The upland soils are generally identified as sinks for CH_4_ (Chan et al., 2001) but additions of biochar and *T. diversifolia* in this experiment reduced the sink capacity (Figs. 1B and 2B). This can likely be attributed to the supply of easily mineralizable C that substitutes for the C oxidized by methanotrophic bacteria (Knoblauch et al., 2008; Zhang et al., 2010). The greatest reduction in CH_4_ uptake, coinciding with urea and high *T. diversifolia* input (Table 4), could be attributed to the availability of NH_4_^+^-N from urea, which is known to inhibit CH_4_ oxidation (Chan and Parkin, 2001, Suwanwaree and Robertson, 2005). Whereas some studies have shown increased uptake of CH_4_ with biochar (Liu et al., 2011, Schimmelpfennig et al., 2014), several have also shown decreases (Spokas et al., 2013; Singla and Inubushi, 2014). This suggests the complexity and variety of factors affecting CH_4_ fluxes in biochar-amended soils. The persistence of biochar effects on fluxes over the two years of observation (Fig. 2B) could be related to improvements in physical properties leading to better drainage and adsorption of dissolved organic carbon (Singh et al., 2012; Zhang et al., 2015) rather than N immobilization discussed above that is likely to be a transient phenomenon.

The consistent reduction in N_2_O with biochar amendments (Figs. 1C and 2C) is similar to previous studies (Rondon et al., 2005, Xiang et al., 2015). The reduction in N_2_O emissions in the present study is lower compared to other studies (such as Zhang et al., 2012a, Zhang et al., 2012b, Zhang et al., 2015) partly because the amounts of added biochar were relatively low (2.5 t ha^−1^ compared with 20–40 t ha^−1^ in all the above studies). N_2_O reduction could have been due to stimulation of microorganisms that can degrade more persistent SOM (Nelissen et al., 2012), resulting in more reactive surfaces. It is also plausible that adsorption of NH_4_^+^ (Taghizadeh-Toosi et al., 2012) and other potential allelochemical inhibitors of microbial metabolic pathways, such as monoterpenes and various polyphenolic compounds that are inhibitory to nitrification, could have played a role (Ball et al., 2011). That the interaction between biochar and urea (Table 3) resulted in reduced N_2_O emission could probably be due to a direct reaction between the biochar surfaces and N_2_O. Thus, the N_2_O emission produced from urea is countered by the biochar. Cayuela et al. (2014) have suggested the catalytic activity of biochar as capable of enhancing the reduction of N_2_O to N_2_. Direct molecular-level interaction between N_2_O and reducing agents has been reported (Hitoshi et al., 2002) and Fungo et al. (2014) observed that steam activation of biochar explained 56% of reduction in N_2_O emission from the same soil. It is plausible that such a reaction could be taking place in biochar amended soils.

The notion that a stimulatory effect of biochar on soil and/or plant respiration levels off during the first years after application (Spokas, 2013) was not seen within the two years of the experiment, as we observed consistent differences in emissions and above ground biomass production during all four cropping seasons. Several studies (e.g. Kimetu et al., 2008, Major et al., 2010, Koide et al., 2014) have shown that biochar contents remain in soil and decreased only between 0.3 and 6% of the amount applied over a period of three years. Whereas some studies (Nelissen et al., 2014) found short-lived effects of biochar (Spokas, 2013), the two-year effects observed in our study support Lentz et al. (2014), who observed a persistent effect over a 3-year period. Lentz et al. (2014) related this multi-year effect to biochar’s physical porosity and chemical binding capacity. The contrasting results in the literature indicate a need for longer-term (>5 years) studies to test this hypothesis.

Results in Fig. 2 show that biochar effects on CH_4_ uptake and N_2_O emissions can persist over two years under field conditions. This could be attributed to the fact that non-aromatic organic materials such as sugars and fats in the pores of biochar (Mukherjee et al., 2015, Koide et al., 2014) are susceptible to mineralization over time (Kim et al., 2011). According to Lentz et al. (2014), the disappearance of these organics with soil aging likely (i) increased porosity and surface area akin to the effect that activation has on charcoal (Fungo et al., 2014); and (ii) increased negatively charged sites on biochar (Cheng et al., 2008). The charged sites can bind NH_4_, making inorganic N only temporarily unavailable for microorganisms and leaching (Dempster et al., 2012), which can later be readily released (Wang et al., 2015). These mechanisms could have been responsible for observed persistence of biochar effect observed in this study. A change in pore size distribution in the biochar-amended soil could have influenced bacterial community composition (Lentz et al., 2014) that could conceivably also affect GHG emissions. Feng et al. (2012) reported decreased ratios of methanogenic to methanotrophic microorganisms that accounted for the decrease in CH_4_ emission from paddy soils. Banerjee et al. (2016) found that moisture affected microbial activity, transcription, composition and ultimately, N_2_O emissions. The reduced N_2_O mitigation effect observed by Spokas (2013) after 3 years of biochar aging could be related to the relatively large particle size (8–40 mm) of the biochar used.

### 4.2 Changes in soil C content

As observed in our study, a previous study carried out in the same area (Kimetu et al., 2008) found that application of biochar increased TOC contents by 6.8 times after adding biochar compared to *T. diversifolia* green manure. In fact, the authors observed that biochar not only remained to a greater extent unchanged, but could be protected by aggregation to a greater degree in soil in the long term. The relatively fine texture of the tested soils means that the soil may allow for more SOC protection by a combination of physical occlusion and organo-mineral interactions.

Chivenge et al. (2011a) found that in clayey soils, the addition of low quality organic resources (such as maize stover) resulted in greater stabilization of SOC and SON in the macro-aggregates, while addition of N-fertilizers enhances their decomposition and faster aggregate turnover leading to less accumulation of SOC and SON. Similar to our results, Chivenge et al. (2011b) also found no interaction between *T. diversifolia* with N fertilizers on similar soils with respect to changes in SOC. This suggests that microbial choice of the N source is independent of the source or that the utilization of freshly applied organic matter (C source) is consumed at the same rate as mineral N (source of N). Verchot et al. (2011) observed that most of the newly added SOC gains from green manures ended up in the coarser aggregates, and is therefore subject to turnover and loss in the event that OM inputs decline. However, using soils from the same experiment, Fungo et al. (2017) found that biochar is stored predominantly as free particulate OC in the silt and clay fraction and promoted a movement of native SOC from larger-size aggregates to the smaller-sized fraction. The increase in C stocks when *T. diversifolia* was added but no additive effect of *T. diversifolia* + biochar may be attributed to the fact that there is a balance between increased mineralization of SOM due to biochar addition on the one hand and an increase in SOC due to greater net primary productivity of root biomass on the other.

Our results (Fig. 3) show that even without returning harvested above ground biomass to the soil, any of the additions were still able to demonstrate SOC gains. It follows that returning maize stover and integrated soil fertility management with biochar can achieve even greater SOC sequestration as well as GHG emission objectives. This implies that soil amendment with organic a mineral resources increases net primary productivity and hence below ground biomass. This biomass can later build up soil organic matter stocks. In terms of climate change mitigation, this is important as it demonstrates the potential for C sequestration associated with soil management.

### 4.3 System C balance

In relatively low fertility soils similar to the one used in this experiment, increases in biomass production in response to biochar application have been widely reported (Major et al., 2010, Li et al., 2015). The grain and biomass yield increase may most likely be attributed in the studied acid soils to increases in direct mineral additions from biochar, improvement in soil physical properties (e.g., bulk density), greater pH and reduced Al toxicity, improved cation exchange capacity, and possibly effects on soil biota that largely remain speculative. The proportional increase in biomass is comparable to other studies on highly weathered tropical soils (Major et al., 2010, Van Zwieten et al., 2010).

The enhanced above ground biomass production that we observed (Table 4) could be due to improved soil physical and chemical properties resulting from biochar amendments. Olmo et al. (2014) found that grain production was correlated significantly and positively with soil moisture, EC, total N, Olsen P and available K, Cu and Zn, and negatively with soil compaction, consistent with the favorable changes in soil physico-chemical properties brought about by biochar additions. According to Vanlauwe et al. (2002) and Lentz et al. (2014), combining biochar with green manure more effectively utilized the two soil amendments, as it eliminates potential plant growth reductions caused by N immobilization after biochar additions and maximized green manure net N mineralization potential.

The increase in net primary productivity due to soil amendments contributes to atmospheric C capture into terrestrial C (above ground and soil organic C). However, it will be necessary for future studies to investigate other types of green manures to better understand limitations over longer periods of time. Application rates used in several other trials are unrealistic for most farming systems. Despite the relatively low application rates of both biochar and *T. diversifolia* used in this study compared to those of previous studies, we demonstrate that significant ecosystem C gains are practically possible in low fertility soils even with relatively moderate amounts of biochar.

The identical grain yields with additions of 2.5 t ha^−1^ biochar and 5 t ha^−1^ tithonia irrespective of urea additions (Table 4) may imply that biochar has the potential to reduce mineral N fertilizer requirements if green manure is added. In terms of GHG emissions, reduced application of mineral fertilizer is one way to reduce the impact of agriculture on climate change. This includes the avoided emissions due to fertilizer production. What remains to be determined is the trade-off between biochar production and fertilizer manufacture in terms of both cost and emissions.

Based on a yield-normalized comparison of our results (EI), biochar can potentially reduce overall GHG emission while improving crop yields and SOC. Nonetheless, the observed reduction in EI due to biochar additions needs to be further analyzed using a full Life Cycle Assessment (LCA) to account for the energy-related emissions needed to produce or transport the biochar among other emissions and emission reductions.

## 5 Conclusions and recommendations

We have shown that the inclusion of biochar in integrated management of low-fertility tropical agricultural soils can reduce GHG emissions and increase ecosystem C gains. The resultant C gains and GHG emission reduction benefits of biochar can be sustained for at least two consecutive years (four seasons) from the time of application under the environmental conditions studied here. This result points to the importance of plant responses for the GHG balance in biochar systems. Our results contribute to mid-term field studies but longer trials might be necessary to better understand the C balance, including different soil types and cropping systems especially where stover biomass is returned to the plot. Longer field-based studies and field studies on soil that show different plant response to organic matter additions are needed to improve understanding of linkages between nutrient use efficiency and GHG mitigation.
